# SlCESTA Is a Brassinosteroid-Regulated bHLH Transcription Factor of Tomato That Promotes Chilling Tolerance and Fruit Growth When Over-Expressed

**DOI:** 10.3389/fpls.2022.930805

**Published:** 2022-07-14

**Authors:** Haiwei Shuai, Tingting Chen, Tanja Wlk, Wilfried Rozhon, Maria J. Pimenta Lange, Tobias Sieberer, Theo Lange, Brigitte Poppenberger

**Affiliations:** ^1^Biotechnology of Horticultural Crops, TUM School of Life Sciences, Technical University of Munich, Freising, Germany; ^2^Institute of Plant Biology, Technical University of Braunschweig, Braunschweig, Germany; ^3^Plant Growth Regulation, TUM School of Life Sciences, Technical University of Munich, Freising, Germany

**Keywords:** BEEs, brassinosteroids, gibberellins, hormones, *Solanum*, SUMOylation

## Abstract

Brassinosteroids (BRs) are required for various aspects of plant growth and development, but also participate in stress responses. The hormones convey their activity through transcriptional regulation and posttranslational modification of transcription factors and one class are basic helix-loop-helix (bHLH) proteins of the BR Enhanced Expression (BEE) subfamily, which in *Arabidopsis thaliana* include BEE1-3 and CESTA (CES). CES and the BEEs promote the expression of different BR-responsive genes, including genes encoding gibberellin (GA) biosynthetic and catabolizing enzymes, as well as cold-responsive genes. Interestingly, in terms of an application, CES could promote both fruit growth and cold stress tolerance when over-expressed in *A. thaliana* and here it was investigated, if this function is conserved in the fruit crop *Solanum lycopersicum* (cultivated tomato). Based on amino acid sequence similarity and the presence of regulatory motifs, a CES orthologue of *S. lycopersicum*, SlCES, was identified and the effects of its over-expression were analysed in tomato. This showed that SlCES, like AtCES, was re-localized to nuclear bodies in response to BR signaling activation and that it effected GA homeostasis, with related phenotypes, when over-expressed. In addition, over-expression lines showed an increased chilling tolerance and had altered fruit characteristics. The possibilities and potential limitations of a gain of SlCES function as a breeding strategy for tomato are discussed.

## Introduction

Brassinosteroids (BRs) are steroid hormones that control many different aspects of plant growth and development, with concentration- and organ-specific effects (Fridman and Savaldi-Goldstein, 2013). In addition, they also take part in adaptive growth processes and in both abiotic and biotic stress responses (Nolan et al., 2020; Ramirez and Poppenberger, 2020). BRs act in concert with other plant hormones and in particular cross-talk with the gibberellins (GAs), has received a considerable amount of research attention in the past few years. BRs and GAs cooperate at the signaling level, but also impact each other’s synthesis and together the hormones jointly control diverse physiological processes including germination, vegetative development in the light and dark, reproductive organ function and fruit development (Bai et al., 2012; Gallego-Bartolome et al., 2012; Li et al., 2012; Stewart Lilley et al., 2013; Bernardo-Garcia et al., 2014; Tong et al., 2014; Unterholzner et al., 2015).

BRs biosynthesis and signaling has been very well-studied in the model plant *Arabidopsis thaliana*. BRs are formed by polyhydroxylation of the sterol campesterol, which requires enzymes of the cytochrome P450s family, such as DWF4, the CYP90s A1-D1 and the CYP85A1 and 85A2, to produce the biosynthetic endproducts castasterone (CS) and brassinolide (BL; Clouse, 2011; Rozhon et al., 2019). CS and BL are both bioactive, as evidenced by their ability to bind the membrane-bound receptor-like kinase BRASSINOSTEROID-INSENSITIVE 1 (BRI1), which, in cooperation with co-receptors, initiates BR signaling (Kinoshita et al., 2005).

BR signaling utilizes posttranslational modification of signaling components for signal-relay. Central factors are GSK3/shaggy-like kinases of the BRASSINOSTEROID-INSENSITIVE 2 (BIN2) subfamily, which phosphorylate BR-controlled transcription factors (TFs) such as members of the BRI1 EMS-SUPRESSOR 1/BRASSINAZOLE-RESISTANT 1 (BES1/BZR1) family and bHLH proteins, including the BES1-INTERACTING MYC LIKEs (BIMs) and BRASSINOSTEROID ENHANCED EXPRESSION 1-3 (BEE1-3), which are closely related to HOMOLOGUE OF BEE2 INTERACTING WITH IBH1 (HBI1) and CESTA (CES; Kim and Wang, 2010; Belkhadir and Jaillais, 2015).

CES, which is also called HALF-FILLED (HAF), is one of 162 members of the typical bHLH protein family of *A. thaliana* [AtbHLH075; (Bailey et al., 2003)]. It is expressed throughout plant development and is present in vascular tissues of roots, hypocotyls and leaves, in leaf axillary meristems, as well as in tissues of the gynoecium, in particular the reproductive tract (Crawford and Yanofsky, 2011; Poppenberger et al., 2011). While CES expression was not seen to be BR-regulated at the transcriptional level, its closest homologues BEE1 and BEE3 were strongly BR-induced (Friedrichsen et al., 2002). An obvious BR-regulation of CES takes place posttranslationally; in response to BRs it is de-phosphorylated and SUMOylated to promote an enrichment in distinct nuclear bodies and increase CES protein stability (Khan et al., 2014).

CES was initially identified as a positive regulator of BR biosynthetic genes (Poppenberger et al., 2011), a function which it shares with the bHLH proteins PHYTOCHROME-INTERACTING FACTOR 4 (PIF4) and HBI1 (Fan et al., 2014; Martinez et al., 2018). In addition, it also promotes the expression of other BR-regulated gene regulons, such as cold-regulated (*COR*) genes, which allows CES to promote freezing tolerance, when over-expressed in *A. thaliana* (Eremina et al., 2016).

Moreover, CES takes part in the control of GA homeostasis. It is required for the BR-induction of the GA biosynthetic gene *GA3ox1* and the GA catabolic gene *GA2ox7* (Albertos et al., 2022). Such dual roles of BR-regulated TFs in the control of both GA biosynthetic and catabolising enzymes have also been reported for *Oryza sativa* (rice), where BZR1 can directly bind to the promoters of the GA biosynthetic genes *GA20ox2* and *GA3ox1*, as well as the GA catabolic gene *GA2ox3*, and induce their expression (Tong et al., 2014). In line with activating a GA catabolic gene, a gain of CES function in the dominant *ces-D* mutant and transgenic plants that over-express CES under the constitutive 35S promoter, showed aspects of phenotypes indicative of reduced GA levels, including delayed flowering, increased leaf axillary meristem activity and an increased GA responsiveness of above ground organs (Albertos et al., 2022). However, in the hypocotyl CES gain of function promoted elongation and during reproductive development, it increased fruit size and promoted fertility in *A*. *thaliana* (Crawford and Yanofsky, 2011; Albertos et al., 2022), speaking for tissue specific effects of CES activity.

Many components of BR biosynthesis and signaling are conserved in crops and BR activity has been very well studied in rice and *Solanum lycopersicum* (tomato), two plant species that originate from subtropical regions. Tomato is a fruit crop with high economic relevance and serves as a model organism for biologists that study fleshy fruit development and cold stress responses of chilling-sensitive plants. Several factors of BR biosynthesis and signaling have been identified in tomato, including the BR biosynthetic enzymes SlCPD and SlDWF4, and the BR signaling components SlBRI1, SlBES1, SlBZR1, and SlBIM1 (Bishop et al., 1999; Koka et al., 2000; Holton et al., 2007; Jia et al., 2021; Mori et al., 2021; Su et al., 2022). Over-expression of *SlBRI1* promoted germination, growth of vegetative and reproductive organs and over-all fruit yield in tomato (Nie et al., 2017; Wang et al., 2019). In addition, and similar to an over-expression of *SlBES1* and *AtBZR1-D*, certain fruit characteristics were altered (Holton et al., 2007; Liu et al., 2014, 2021). Over-expression of the bHLH protein *SlBIM1* on the other hand, whose orthologues act as positive regulators of growth in *A. thaliana*, reduced plant size and fruit yield in tomato (Yin et al., 2005; Mori et al., 2021), showing that the outcomes of over-expressing BR signaling components can be species-specific and are not predictable, based on sequence similarities only.

Here it was aimed to identify a CES orthologue of tomato, to assess if it can be utilized to promote cold stress tolerance and fruit growth through over-expression. Based on amino acid sequence similarity and the presence of regulatory motifs, SlCES was identified and the effects of its hyper-accumulation in tomato were analysed. This showed that aspects of CES function are conserved in tomato and that increasing *SlCES* expression merits to be considered as a breeding strategy for tomato.

## Materials and Methods

### Plant Material, Growth Conditions and Phenotypic Assessments

*Solanum lycopersicum* ecotype Heinz 1706 was the genetic background used in this study. For seedling phenotyping, seeds were germinated in pots containing the soil substrate SPED63P (Patzer GmbH, Sinntal-Altengronau, Germany) and grown in a growth chamber (Bright Boy; CLF Plant Climatics, Wertingen, Germany) set to standard growth conditions of 21 ± 2°C and 16 h white light/8 h dark cycles, with a light intensity of 80 μmol m^–2^s^–1^.

GA treatments were performed by spraying the seedlings with water containing 50 μM GA_4+7_ (Duchefa, Haarlem, NL) until dripping wet. The GA treatments were started at 7 days post germination and were done daily for 7 days, before hypocotyl and epicotyl elongation was measured.

For phenotyping in the adult stage, the plants were pre-grown in the incubators and transferred to the greenhouse when they were 2 weeks old. Phenotyping of vegetative growth parameters in the greenhouse was conducted between August and October in Freising (Germany; 48° 24′ N, 11° 45′ O) without artificial lightning and at a temperature of 25 ± 3°C for standard growth conditions or 18 ± 3°C for low temperature phenotyping.

For fruit phenotyping fully ripe fruits were harvested twice a week and were weighed. For a documentation of their morphology, representative fruits from each developmental stage were selected and photographed. For fruit phenotyping at low ambient temperature, all fruits that had developed at least to the green stage were counted at different time points.

To determine seed characteristics, seeds of ripe fruits were placed into a beaker and covered with film (Parafilm, Sigma-Aldrich, United States) and were incubated at room temperature for 24 h, before the gel and pulp was removed. The seeds were then washed, air-dried on two layers of filter paper, and 100 seeds were weighed in replicates from different plants, to determine the 100-seed-weight.

### *Solanum lycopersicum* bHLH Protein Identification and Phylogenetic Analysis

To identify a CES orthologue in the *S. lycopersicum* genome, the NCBI database was searched for bHLH proteins. This identified a number of candidates, from which the bHLH domain was extracted with the Pfam function of the Simple Modular Architecture Research Tool (SMART, Heidelberg). The bHLH domain was then used as a query sequence and multiple blast searches were run in the *S. lycopersicum* databases of the NCBI, the TIGR Plant Transcript Assemblies and Sol Genomics Network (SGN). This retrieved 90 bHLH domains that were used for multiple blastp searches in the ITAG RELEASE 2.3 PREDICTED PROTEINS (sl2.4) SGN database with the expect (e-value) threshold and the maximum hits set to 10 and 10,000, respectively. The acquired protein identifiers were extracted, and their sequences were obtained from SGN. All sequences were then analyzed with three independent protein structure analysis databases, including the SMART, Pfam and Conserved Domain Database. Based on these analyses, proteins without a bHLH domain, or identified as TCP proteins, were removed. After this final selection, 166 genes remained. These were compared with the genes published by Sun et al. (2015) and Wang et al. (2015), resulting in a final list of 168 members of the tomato bHLH family.

Phylogenetic analyses were conducted in MEGA7 (Kumar et al., 2016). The sequences of the identified 166 tomato bHLH proteins together with the published 162 *Arabidopsis* bHLH proteins were aligned with the ClustalW Multiple alignment method in MEGA7. The phylogeny was inferred using the Neighbor-Joining method. The percentage of replicate trees in which the associated taxa clustered together in the bootstrap test (1000 replicates) was calculated. The evolutionary distances were computed using the number of differences method.

### Generation of SlCES Constructs and Transgenic Lines

To clone the *35S:SlCES-YFP* construct, the coding sequence of *Solyc12g036470.1.1* was PCR-amplified from tomato cDNA and cloned upstream and in frame with YFP into vector pGWR8, which contains a kanamycin resistance marker (Rozhon et al., 2010). Mutations were introduced into the lysines (K) of two putative SUMOylation sites in SlCES (K126 and K242), by site-directed mutagenesis, using primers with integrated nucleotide changes that yield a replacement of the K with arginines (R), generating 35S promoter-driven YFP fusions with the SlCES^K126R^ and SlCES^K242R^ single mutants. The double mutant reporter was generated by fusing N-termini and C-termini of the two SlCES single mutants, resulting in a *35S:SlCES-YFP*^K126R+K242R^ construct. These constructs were used for protoplast transformation.

For the generation of transgenic plants that stably express *35S:SlCES-YFP*, the KAN resistance marker in pGWR8 was replaced with a *BIpR* gene from pGreenII0229 (Mallory et al., 2005), to generate a transgene that confers glufosinate-resistance. Transgenic lines were generated by Agrobacterium-mediated leaf-disc transformation using *Agrobacterium tumefaciens* strain GV3101. Individuals homozygous for the *35S:SlCES-YFP* transgene were selected based on the glufosinate resistance and using genotyping.

### Quantitative Real-Time PCR

For Quantitative Real-Time PCR (qPCR), young leaves from 12-week-old plants grown in greenhouse at normal ambient temperature were harvested and frozen in liquid nitrogen for further experiments. Total RNA was extracted with the E.N.Z.A. Plant RNA Kit (OMEGA Bio-tek, GA, United States) and treated with DNase I (Thermo Scientific, MA, United States). 1 μg of RNA was used for cDNA synthesis with the RevertAid first-strand cDNA synthesis kit (Thermo Scientific, MA, United States). qPCRs were performed with an Eppendorf realplex^2^ Mastercycler (Eppendorf, Hamburg, Germany) using the SensiFAST SYBR Lo-ROX Mix (Bioline, London, United Kingdom) and the primers YFP forward (5′-AAGCAGAAGAACGGCATCAA-3′) and YFP reverse (5′-GGGGGTGTTCTGCTGGTAGT-3′), to amplify parts of YFP. Data were normalized to *SlACT* and samples were measured in at least three biological and four technical replicates.

### Protoplast Transformation and Fluorescence Microscopy

Protoplast were generated from leaves of 30-day-old plants, grown under sterile conditions on 1/2 MS medium (Duchefa, Haarlem, NL) solidified with 0.7% w/v agar (Duchefa, Haarlem, NL). Protoplasting of mesophyll cells was performed from leaf strips, with a cellulase-based cell wall digestion. The protoplasts were then transformed with the 35S:SlCES-YFP wild-type and mutant constructs, with a polyethyleneglycol-mediated transformation protocol, using the same methodology as described previously for *A. thaliana* (Khan et al., 2014).

Following protoplast transformation, SlCES-YFP wild-type and mutant localization was analyzed with or without treatment with 1 μM 24-epiBL or bikinin for 2 h, (as described previously in Khan et al., 2014), with a Leica TCS-NT confocal microscope (Leica, Weltzlar, Germany). YFP-tagged fusion proteins were excited using the Ar laser line at 514 nm and detected at 520–550 nm. The images were assembled with the Leica confocal software LSC Lite version 2.61.

### Gibberellin Measurements With Gas-Chromatography-Mass Spectrometry

Quantitative analysis of endogenous GAs was performed from aerial parts of 2-week-old plants, grown in soil, in the incubator run at the standard conditions described above. GAs were measured in the samples using gas-chromatography-mass spectrometry (GC-MS) as described previously (Lange et al., 2020). The ions monitored for quantification of endogenous GAs were 300 and 302 (GA_12_), 448 and 450 (GA_53_), 432 and 434 (GA_44_), 434 and 436 (GA_19_), 418 and 420 (GA_20_), 447 and 449 (GA_29_), 448 and 450 (GA_1_), 594 and 596 (GA_8_), 270 and 272 (GA_12_-ald.), 298 and 300 (GA_110_), 239 and 241 (GA_15_), 314 and 316 (GA_24_), 298 and 300 (GA_9_), 268 and 270 (GA_51_), 418 and 420 (GA_4_), and 506 and 508 (GA_34_).

### Profiling of Amino Acids, Titratable Acids and Ions in Tomato Fruits

The measurements of metabolites were done from fruits at the green, breaker and ripe stages, which were harvested at the same time point from plants grown at normal ambient temperatures of 25 ± 3°C. For the analyses, for each stage 4 representative fruits were harvested from each plant (5 wild type and 5 *35S:SlCES-YFP* #9 plants), were pooled and homogenized using a waring blender mixer.

For analysis of minerals, 4 g homogenate were weighed into a quartz crucible, dried at 105°C and finally incinerated at 550°C for 5 h. After cooling, 5 ml 20% nitric acid was added to the crucible and the liquid evaporated at 100°C to dryness. The residue was dissolved in 5 ml 1 M nitric acid and water added to a final volume of 25 ml. Potassium was measured directly while for determination of calcium lanthanum (III) nitrate was added to a final concentration of 1% prior measurement using a Jenway PFP7 flame photometer (Cole-Parmer, Staffordshire, United Kingdom).

For determination of titratable acid and amino acids the homogenate was centrifuged and filtered. Titratable acid was determined by titration with 0.1 M sodium hydroxide to a pH of 8.0. For amino acid profiling a LC-10 high-pressure gradient HPLC system equipped with a RF-10Axl fluorescence detector (Shimadzu, Kyoto, Japan) and a Nucleodur 100-5 C18ec 250 × 4 mm HPLC column (Machery-Nagel, Düren, Germany) was used.

Samples were spiked with L-2-aminoadipic acid as internal standard. Aliquots (10 μl, 1:100 diluted) were automatically derivatized by the autosampler by addition of 70 μl OPDA-reagent (85 μl o-phthalaldehyde 20 mg/min in methanol, 1.7 μl 3-mercaptopropionic acid and 1615 μl 50 mM sodium carbonate buffer pH 9.5) and incubation for 2 min. Subsequently, 10 μl 1 M formic acid was added and 5 μl of the final mixture injected into the HPLC system. Eluent A consisted of 20 mM potassium dihydrogenphosphate containing 30 ml/l tetrahydrofurane and set with potassium hydroxide to 7.25. Eluent B consisted of 45% (v/v) ACN, 45% (v/v) methanol and 10% (v/v) water. Gradient elution was performed at a flow rate of 0.7 ml/min and started with 100% A for 1 min. Subsequently, the concentration of B was raised to 30% within 19 min, then within 10 min to 34%, then within 7 min to 62% and finally within 1 min to 90% prior returning it to the initial conditions (100% A and 0% B). The column was re-equilibrated for 7 min prior injection of the next sample. The column oven was set to 35°C and the fluorescence detector to an excitation wavelength of 350 nm and an emission wavelength of 450 nm. Chromatograms were evaluated with the Clarity software package (DataApex, Prague, Czech Republic).

## Results

### *Solyc12g036470.1.1* Encodes the bHLH Protein SlCES, Which Enriches in Nuclear Bodies in Response to BR

To identify CES orthologues in the tomato genome, the bHLH domain sequence was used as a query, and multiple tblastn searches were performed in the databases of NCBI, SGN and TIGR. With this approach, 90 bHLH-like domains of tomato were obtained. With their bHLH domain sequences, the ITAG RELEASE 2.3 PREDICTED PROTEINS (sl2.4) database of SGN was searched. In order to remove genes that do not belong to the bHLH family, the identified tomato genes were checked with SMART, Pfam and Conserved Domain Database. This yielded 166 proteins as putative members of the tomato bHLH family, which were given chronological identifiers based on their position on the chromosomes (Supplementary Table 1). In two previous studies, that had also investigated the bHLH family of *S. lycopersicum* 152 and 159 members had been identified (Sun et al., 2015; Wang et al., 2015). A summary of all identified tomato bHLH proteins, comparing the results of this and the two previous studies is shown in Supplementary Table 1. With the approach used in this work one bHLH protein was missed, making it a total of 168 bHLH family members in *S. lycopersicum*.

After aligning the Arabidopsis bHLH proteins (Heim et al., 2003) with the identified tomato bHLH-type proteins using the ClustalW Multiple alignment method, their phylogenetic relationship was analyzed. A phylogenetic tree was constructed with the Molecular Evolutionary Genetics Analysis (MEGA) software version 7 (Kumar et al., 2016) using the neighbor-joining method. In this phylogenetic tree, three close *CES* orthologues of tomato were identified, namely SlbHLH062 (*Solyc04g007300.2.1*), SlbHLH054 (*Solyc03g119390.2.1*) and SlbHLH160 (*Solyc12g036470.1.1*; Supplementary Figure 1).

**FIGURE 1:**
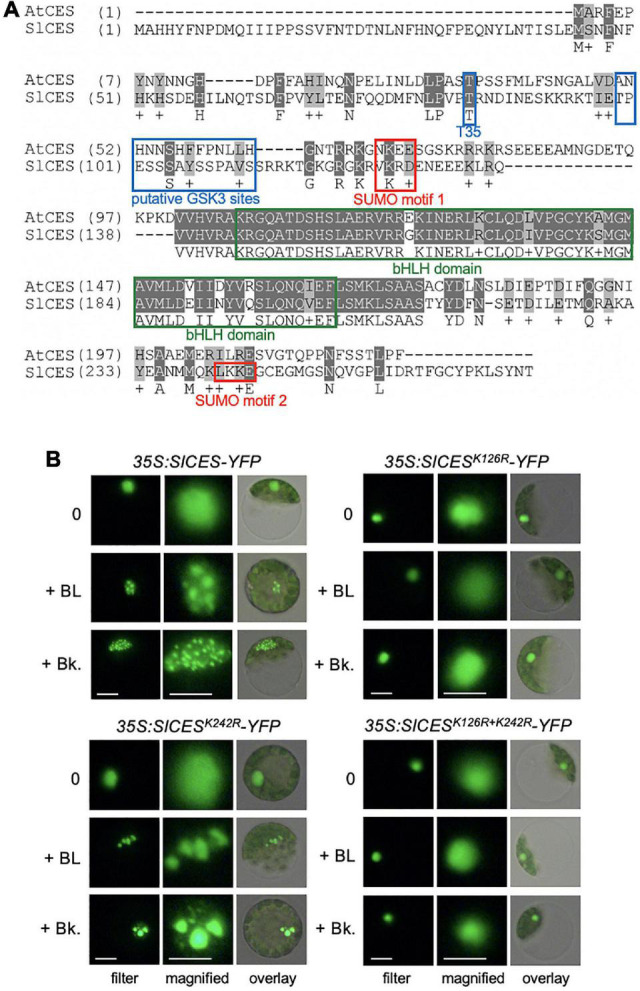
*Solyc12g036470* encodes a CES orthologue, which enriches in subnuclear domains in response to BR. **(A)** Amino acid sequence alignment of CES of *A. thaliana* (AtCES) and CES of *S. lycopersicum* (SlCES). Motifs with functional relevance in AtCES are highlighted. **(B)** SlCES-YFP wild-type and SUMO mutant versions were expressed under control of the 35S promoter in tomato mesophyll protoplasts, which were either left untreated (0) or treated with 1 μM of the BR 24-epibrassinolide (+ BL) or 1 μM of bikinin (Bk). The images show CES-YFP localization in representative protoplasts, analyzed and imaged with fluorescence microscopy. White scale bars: filter = 10 μm, magnified = 6 μm.

To address, if the identified tomato bHLH proteins may contain motifs that are of functional relevance for CES modes of activity in *A. thaliana*, amino acid sequence alignments were assembled, which showed that a BIN2 recognition site, positioned at T35 in AtCES (Khan et al., 2014), and a SUMOylation motif were conserved in SlbHLH160, but not in the other two candidates (Figure 1A). Moreover, SlbHLH160 also contained 2 classical GSK3/shaggy like motifs [S/TxxxS/T; (Frame and Cohen, 2001)], positioned between T99 and S112 and a second SUMOylation motif from L241-E244.

In light of the presence of these putative regulatory motifs, it was of interest to investigate whether the subcellular localization of this CES orthologue SlbHLH160, which was named SlCES, may be controlled by BR. A reporter construct consisting of full length SlCES fused to YFP and driven by CaMV 35S promoter was created and transformed into tomato protoplasts. Under non-induced conditions, SlCES-YFP localized diffusely to the nucleus. However, after 2 h of treatment with BL or the BIN2-inhibitor bikinin, which also activates BR signaling (De Rybel et al., 2009), SlCES-YFP was enriched in nuclear bodies (Figure 1B). The size, number and shape of SlCES nuclear bodies was highly variable and this is in analogy to AtCES-containing nuclear compartments (Khan et al., 2014).

Therefore, *Solyc12g036470.1.1* encodes the bHLH protein SlCES, which contains BR-regulatory motifs and is controlled in its subnuclear localization by BRs.

### A Conserved SUMOylation Site Is Required for BR-Induced Nuclear Re-Localization of SlCES

BR-induced subnuclear compartmentalization of AtCES is enabled by SUMOylation (Khan et al., 2014). To address if, this may also be the case for SlCES, the lysines (K) of the two typical SUMOylation consensus motifs [defined by ΨKX(E/D); (Gareau and Lima, 2010)] were mutated to arginines (R) by site directed mutagenesis. The resulting SlCES^*K*126*R*^ and SlCES^*K*242*R*^ single mutants, as well as a double mutant combining both mutations, were fused to YFP and transformed into tomato protoplasts. The protoplasts were then treated with either BL or bikinin and analyzed with fluorescence microscopy. This revealed that mutation of K126 rendered SlCES nuclear localization unresponsive to BL and bikinin treatment (Figure 1B). Mutation of K242 had no effect, as shown using a the SlCES^*K*242*R*^ single and a SlCES^*K*126*R+K*242*R*^ double mutant, providing evidence that K126, an amino acid embedded in a conserved SUMOylation motif, is required for BR-induced nuclear compartmentalization. Thus, SlCES is a protein that re-localizes to subnuclear domains in response to an activation of BR signaling, an ability that, like in AtCES, requires the lysine of a conserved SUMOylation motif.

### *SlCES* Over-Expression Alters GA Homeostasis

**FIGURE 2:**
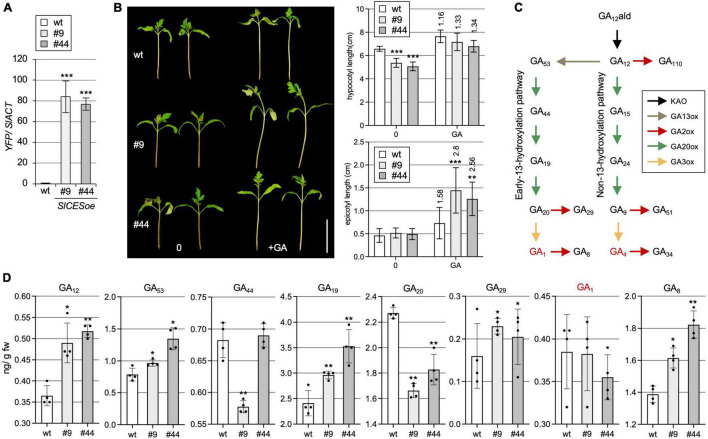
*SlCES* over-expression alters GA homeostasis. **(A)** Expression of *SlCES-YFP* in two independent over-expression lines (#9 and #44) as compared to wild-type Heinz 1706 (wt). *YFP* was detected by RT-qPCR in vegetative tissues of the lines. Data show the mean ± SD. *n* = 4 biological repeats, each measured in 3 technical replicates, normalized to *SIACT*. **(B)** Phenotype (left) and quantification of hypocotyl and epicotyl length (right) of 14-day-old, soil-grown plants of the two SlCESoe lines with or without GA treatment. Plants were grown in the incubator in standard growth conditions for 7 days and then treated with DMSO as a control (0), or with 50 μM GA_4+7_ (GA), once a day for another 7 days, before hypocotyl and epicotyl length was measured. The results are shown as means ± SD (*n* > 12). The ratio of treated versus untreated is shown. White bar = 5 cm. **(C)** Illustration of the GA biosynthetic pathway. Different enzyme classes are shown in different colors. **(D)** GC-MS measurements of different GAs in aerial parts of 14-day-old plants grown in the incubator in the same conditions as in A. The values are given in ng/g fresh weight. The results of four biologically replicates and mean and standard deviations are shown. In all charts: asterisks indicate significant differences determined by Student’s *t*-test (*P ≤ 0.05; **P ≤ 0.01; ***P ≤ 0.001).

Given these features, there was evidence that SlCES is a AtCES orthologue. Therefore, to investigate if aspects of CES activity are conserved in tomato, *SlCES* over-expression lines were generated. To do so, the “Heinz 1706” genetic background, which was used for whole genome sequencing (Tomato Genome, 2012), was transformed with a *35S:SlCES-YFP* construct. Two independent transgenic lines, homozygous for the transgene, with high expression levels, SlCESoe-9 and SlCESoe-44 (Figure 2A), were selected and their seedling phenotype was analyzed. This showed that hypocotyl elongation was significantly reduced (Figure 2B). Since AtCES can induce GA catabolism, we tested, if this reduced hypocotyl elongation may relate to GA shortage. Therefore, we treated the plants with GA, which showed that the hypocotyl elongation defects in both over-expression lines were alleviated through GA application. Moreover, the experiment showed that SlCESoe-9 and SlCESoe-44 seedlings were hyper-responsive to GA, since the epicotyls (the stem section between the cotyledons and the first true leaf pair) of both lines elongated significantly more strongly than wild-type in response to the GA treatment (Figure 2B).

To reveal, if the over-expression lines have defects in GA biosynthesis, the levels of different GA intermediates, both from the non-and the early C13-hydroxylation pathway [Figure 2C; (Hedden, 2020)] were determined in seedlings by GC-MS. Interestingly, in SlCESoe-9 and SlCESoe-44 plants, there were clear differences to wild-type in GA-levels of the early C13 hydroxylation pathway (leading to GA_1_), while the non-C13-hydroxylation pathway (leading to GA_4_) was not significantly affected (Figure 2D and Supplementary Figure 2). The precursor for both routes, GA_12_, was significantly increased, and the level of GA_53_, the first precursor of the early C13-hydroxylation pathway, which is derived directly from GA_12_, was also significantly increased. However, GA_20_, a direct product of GA_53_ was significantly decreased in both over-expression lines, indicating a reduced activity of a GA20-oxidase (GA20ox) enzyme, which catalyzes this reaction. While, somewhat surprisingly, levels of the bioactive GA GA_1_, a direct product of GA_20_, were not found to be significantly altered, levels of its catabolite GA_8_ were significantly increased (Figure 2D), speaking for increased activity of a GA2-oxidase (GA2ox) that catalyzes conversion of GA_1_ into GA_8_. In line with increased GA2ox activity, also GA_29_ levels were slightly increased, even though its substrate GA_20_ was depleted in the SlCES over-expression lines (Figure 2D), again indicates strong GA2ox activity. Thus, SlCES over-expression has clear effects on the early C13 hydroxylation pathway of tomato.

### *SlCES* Over-Expression Improves Growth and Fruit Set at Low Ambient Temperatures

**FIGURE 3:**
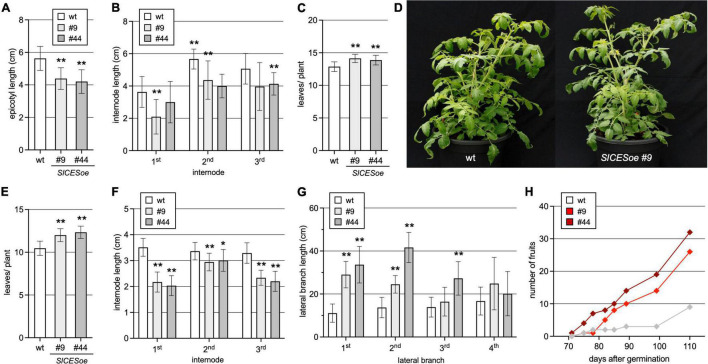
Over-expression of *SlCES* promotes lateral branch growth and fruit set in low ambient temperatures. **(A–D)** *SlCES* over-expressing plants and wild-type at normal ambient temperatures of 25 ± 3°C. The plants were grown in soil, in long-day conditions in the greenhouse. Different growth parameters were assessed from 15 plants of each genotype, including epicotyl length after 5 weeks **(A)**, and the length of the first three internodes **(B)** and the number of leaves **(C)** after 8 weeks. Photos of representative plants at 7 weeks post germination are shown **(D)**. **(E–H)** *SlCES* over-expressing plants and wild-type at low ambient temperatures of 18 ± 3°C. The plants were grown at lower temperatures, but with otherwise comparable conditions as in panel **(A–D)** and the following growth parameters were assessed: leaves per plant **(E)**, the length of the first three internodes **(F)** and the length of the first 4 lateral branches **(G)**, all after 8 weeks of development. In addition, the total number of fruits that had developed at least to the green stage was counted at different stages of development from 15 plants per line **(H)**. The results in all bar charts are shown as means ± SD (*n* = 15). (*P < 0.05; **P < 0.01; Student’s *t*-test).

In Arabidopsis CES can increase freezing stress tolerance when over-expressed and this is due to its ability to induce the C-repeat/DRE-Binding Factors (*CBFs)*, but also non-CBF pathways (Eremina et al., 2016) implicated in cold tolerance. Since tomato is a chilling-sensitive plant and has hardly any capacity to cold acclimate (Zhang et al., 2004), it was of interest to test if SlCES may be able to impact pathways that confer tolerance also to chilling conditions, which cause damage by different means (Ramirez and Poppenberger, 2020). To investigate this, wild-type and the SlCESoe-9 and SlCESoe-44 lines were first grown at normal ambient temperatures of 25 ± 3°C. This showed that SlCES over-expression did not induce obvious morphological defects. However, when growth parameters were quantified, it was found that it had a repressive effect on stem elongation in earlier stages of adult plant development (weeks 4-8), as evidenced by shorter epicotyls and shorter internodes (Figures 3A,B). The number of leaves of the SlCESoe-9 and SlCESoe-44 lines was increased as compared to wild-type, resulting in very subtle growth changes (Figures 3C,D).

When the plants were grown at a low ambient temperature of 18 ± 3°C, the differences in the number of leaves and internode length to wild-type became more pronounced (Figures 3E,F). Furthermore, in the two over-expression lines the 1*^st^* and 2*^nd^* lateral branches developed significantly better (Figure 3G). Moreover, while in these growth conditions wild-type still flowered, fruit development was strongly suppressed. However, fruit set in plants of the SlCESoe-9 and SlCESoe-44 lines was much less affected, resulting in a higher number of fruits at low temperatures (Figure 3H).

### SlCES Over-Expression Impacts Growth and Metabolite Composition of Tomato Fruits

**FIGURE 4:**
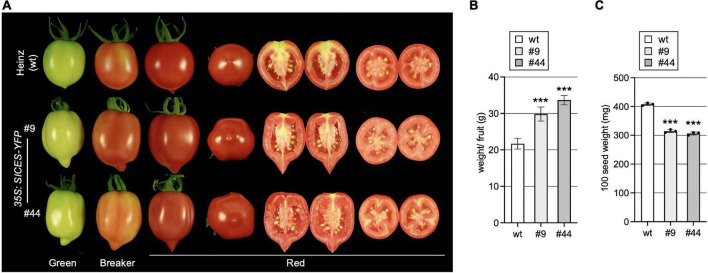
*SlCES* over-expression alters fruit development. **(A)** Photos of representative fruits of *SlCESoe* plants and wild-type at different developmental stages. For red fruits transverse and longitudinal sections are shown. **(B)** Average fruit weight of fully ripe fruits of *SlCESoe* plants. 10–15 fully ripe fruits were harvested and weighed to obtain a total weight, which was then divided by the fruit number, to obtain a mean. Data show the mean ± SD of *n* = 6 (***P < 0.001; Student’s *t*-test). **(C)** Quantification of 100-seed-weight. Seeds were collected from ripe fruits, air-dried and 100 seeds were weighted. Data show the mean ± SD of *n* = 3 (***P < 0.001; Student’s *t*-test).

AtCES improves fertility by promoting extracellular matrix production and induced cell death in the reproductive tract, which is required for efficient pollen tube elongation. In addition, it also promotes growth of *A. thaliana* fruits, which are siliques with two fused carpels, leading to longer fruits with bent tips, when AtCES is ectopically expressed (Crawford and Yanofsky, 2011). To study if this function is conserved in fleshy fruit development, we assessed fruit growth in the two SlCES over-expressing lines. This showed that SlCESoe-9 and SlCESoe-44 transgenic plants formed longer fruits with more strongly pointed and slightly bent ends, a more cylindric shape and an increased weight as compared to those of wild-type Heinz 1706 (Figures 4A,B). While seed set was increased, the seeds of SlCES over-expressing plants were significantly smaller than those of wild-type, which we quantified by weighing them (Figure 4C).

To asses fruit quality characteristics, different ions, amino acids and titratable acids were measured in green, breaker stage and ripe fruits of the SlCESoe-9 line and wild-type, which showed that calcium levels were significantly increased, whereas titratable acid levels were decreased throughout fruit development in the over-expression line. Moreover, the concentration of the amino acids asparagine, aspartic acid, glutamic acid, threonine, arginine and tryptophan were decrease in early stages of fruit development (green fruits) in SlCES over-expressing plants (Table 1). In the red stage the differences changed, with aspartic acid, serine, alanine and tyrosine being increased in ripe fruits of the SlCES over-expression line. Thus, in summary, SlCES, promoted growth and changed the amino acid profile of developing fruits, but repressed seeds size, when over-expressed in *S*. *lycopersicum*.

**TABLE 1:** Concentration of relevant minerals and amino acids in fruits of SlCESoe-9 and wild-type plants at the green, breaker, and ripe stages.

		WT			SlCESoe-9		
		Green	Breaker	Red	Green	Breaker	Red
Potassium	mg/kg FW	3734.3 ± 255	3394.6 ± 260	3326.5 ± 414	3415.6 ± 237	3072.4 ± 246	3203.0 ± 384
Calcium	mg/kg FW	87.4 ± 15	78.7 ± 7	88.1 ± 10	117.8 ± 24*	135.6 ± 24^**^	142.1 ± 28^**^
Phosphorous	mg/kg FW	411 ± 53	351 ± 28	353 ± 37	373 ± 15	352 ± 69	368 ± 44
Titratable acid	mmol_*eq*_/l	119 ± 6	101 ± 15	62 ± 7	90 ± 12^**^	83 ± 7	52 ± 4*
NH_4_	mg/l	573 ± 113	408 ± 66	312 ± 33	626 ± 61	437 ± 61	341 ± 61
GABA	mg/l	902 ± 162	696 ± 56	532 ± 49	1021 ± 130	744 ± 128	593 ± 113
Total free amino acids	mg/l	5747 ± 232	6108 ± 932	5756 ± 662	4939 ± 479*	5630 ± 1504	6927 ± 998
Asparagine	mg/l	1128 ± 166	929 ± 211	528 ± 76	826 ± 203*	627 ± 187*	558 ± 98
Aspartic acid	mg/l	146 ± 141	368 ± 52	548 ± 79	43 ± 36	345 ± 40	757 ± 113*
Glutamic acid	mg/l	308 ± 27	1232 ± 223	1903 ± 319	214 ± 9^***^	1011 ± 132	2187 ± 313
Serine	mg/l	90 ± 13	61 ± 12	60 ± 13	111 ± 30	68 ± 16	89 ± 12^**^
Glutamine	mg/l	2822 ± 137	2592 ± 362	2049 ± 150	2586 ± 330	2340 ± 417	2508 ± 394
Histidine	mg/l	101 ± 9	99 ± 18	86 ± 12	90 ± 18	79 ± 26	102 ± 22
Glycine	mg/l	19 ± 4	30 ± 7	43 ± 7	32 ± 15	28 ± 7	51 ± 10
Threonine	mg/l	149 ± 12	127 ± 20	101 ± 13	128 ± 15*	106 ± 28	123 ± 23
Arginine	mg/l	185 ± 64	124 ± 46	57 ± 19	87 ± 16*	79 ± 44	54 ± 15
Alanine	mg/l	50 ± 13	27 ± 4	61 ± 18	71 ± 18	28 ± 6	120 ± 18^***^
Tyrosine	mg/l	130 ± 9	76 ± 21	36 ± 7	119 ± 18	66 ± 36	50 ± 5*
Valine	mg/l	83 ± 15	45 ± 16	26 ± 6	112 ± 31	63 ± 42	34 ± 6
Methionine	mg/l	14 ± 2	15 ± 5	21 ± 4	16 ± 6	13 ± 4	25 ± 3
Tryptophan	mg/l	33 ± 7	27 ± 7	21 ± 4	20 ± 7*	19 ± 11	21 ± 3
Phenylalanine	mg/l	245 ± 30	171 ± 41	106 ± 18	245 ± 26	166 ± 49	124 ± 24
Isoleucine	mg/l	79 ± 7	60 ± 18	28 ± 8	93 ± 21	64 ± 27	35 ± 10
Leucine	mg/l	75 ± 7	60 ± 20	31 ± 9	78 ± 22	56 ± 22	35 ± 10
Lysine	mg/l	91 ± 41	65 ± 13	50 ± 9	68 ± 11	63 ± 22	53 ± 8

Data show the mean ± SD of n = 5. Significant changes are marked in red (*P < 0.05; ^**^P < 0.01; ^***^P < 0.001; Student’s t-test).

## Discussion

BRs are plant hormones that have the capacity to promote growth and at the same time increase resistance against certain abiotic stress types, including cold stress. This makes them interesting for plant production and BR-containing substances are used as growth stimulants in agriculture (Vriet et al., 2012). Moreover, factors that control BR responses are breeding targets in crops such as rice, maize and barley (Castorina and Consonni, 2020) and thus their functional analysis can benefit breeding. However, as yet most of our understanding of BR modes of action comes from *A. thaliana* and it is therefore important to test for the transferability of findings from this model plant to crops, to understand possibilities and limitations of their application.

*S. lycopersicum*, the cultivated tomato, is the worlds’ most important fruit crop. As a plant species that originates from subtropical climates it has a high temperature requirement for optimal yield and lacks abilities to tolerate frost. Chilling injuries can occur already at a temperature of 12°C and temperatures below 19–20°C are suboptimal for its performance (van Ploeg and Heuvelink, 2005). Therefore, an important aim of research is to identify factors that can increase the chilling tolerance of this crop and since CES can improve cold stress tolerance and fruit development in *A. thaliana*, we wanted to test if these abilities are conserved in tomato.

An amino acid homology-based BLAST search of the *S. lycopersicum* genome identified 166 bHLH proteins, which differed to previous studies that had identified 152 or 159 members (Sun et al., 2015; Wang et al., 2015). Pooling the proteins of this and the two previous studies made it a total of 168 bHLH proteins in tomato. A phylogenetic comparison of the bHLH proteins of tomato and *A. thaliana*, identified three tomato bHLH proteins with high sequence similarities to the CES/BEE bHLH subfamily. However, motifs with functional relevance for CES activity in *A*. *thaliana* were present only in the protein encoded by locus *Solyc12g036470*. The protein was named SlCES and while it was expressed diffusively in the nucleus in standard conditions, it re-localized to subnuclear domains, when tomato protoplasts were treated with BL or bikinin. This ability depended on a conserved SUMOylation site, which confers BR-induced nuclear compartmentalization also in *A. thaliana* (Khan et al., 2014), showing that protein features that allow for a BR regulation are conserved in SlCES and providing first evidence that SUMOylation of BR signaling components occurs in tomato.

SlCES was over-expressed in tomato and repressed hypocotyl elongation in seedlings, a phenotype that could be rescued with external GA. Other phenotypes of SlCES over-expressing plants, like reduced internode elongation and a GA hyper-responsiveness also indicated reduced levels of bioactive GA and, when GAs were measured, it was found that in aerial tissues of whole seedling, the levels of several intermediates of the early C13-hydroxylation pathway, which forms GA_1_, were altered. GA_53_ levels were increased, while GA_20_ levels were significantly decreased; the intermediates GA_44_ and GA_19_ were not consistently changed in both SlCESoe lines. Given the GA-deficient phenotypes of the SlCESoe lines, it was surprising that GA_1_, a direct product of GA_20_, was not altered, whereas its catabolite GA_8_ was significantly increased. The increased GA_8_ levels may indicate an increased activity of a GA 2-oxidase of class I or II, which directly convert C_19_ GAs such as GA_20_ and GA_1_ to GA_29_ and GA_8_, respectively (Hedden, 2020; Lange and Pimenta Lange, 2020). In *A. thaliana* AtCES induces GA catabolism by increasing expression of the class III GA 2-oxidase GA2ox7, which converts GA_12_ to GA_110_ (Lange et al., 2020). However, since GA_110_ levels were not altered in seedlings of the SlCESoe lines, our data suggest that in the analyzed tissues SlCESoe induces class I and/or II GA2oxs rather than class III GA 2-oxidases and these differences may be caused by differences in the presence of regulatory motifs and/or co-factors required for CES activity.

An increased activity of GA 2-oxidases that synthesize GA_8_ should deplete GA_1_, however, GA_1_ was not significantly decreased in the SlCESoe lines, albeit a slight tendency to reduced levels was seen in line SlCESoe-44. This may be due to technical limitation of the GA analytics, since all aerial plant parts were measured and thus cell-type specific differences in GA_1_ levels may not have been revealed. Also, in the measured tissues and at this developmental stage, the increased conversion of GA_1_ to GA_8_ may have been compensated for. This could occur via an induction of GA 3-oxidase activity, and the clear reduction of the GA 3-oxidase substrate GA_20_ supports this idea. Since in *A. thaliana*, CES over-expression also induced GA 3-oxidase activity, there is clear evidence, that the complex roles of CES in the control of both GA biosynthesis and GA catabolism are conserved in tomato.

In *A. thaliana*, CES over-expression produced phenotypes indicative of GA deficiency during the vegetative growth phase of adult plants, however, in hypocotyls of seedlings and also in fruits growth was promoted, speaking for tissue and developmental stage specific outcomes (Crawford and Yanofsky, 2011; Albertos et al., 2022). In tomato, SlCES over-expression suppressed hypocotyl elongation, a phenotype that is expected if GA levels are reduced, but was not seen in *A*. *thaliana*, where the GA deficiency in hypocotyls of *ces-D* appears to be masked by other effects (Albertos et al., 2022). However, in analogy with AtCES over-expression, SlCES promoted fruit growth, yielding larger, more elongated fruits, with bent tips in the SlCESoe lines. Such fruits formed more, but smaller seeds, a phenotype that was not reported in *A. thaliana* (Crawford and Yanofsky, 2011). Whether the larger fruits in AtCES and SlCES over-expressing plants are cause by increased mitotic activity or more cell expansion is currently unknow, and it will be interesting to analyze this in future.

When different primary and secondary metabolites were analyzed in fruits of SlCES over-expressing plants, it was found that their metabolite profile was altered. They hyper-accumulated calcium, but showed reduced concentrations of titratable acids and the amino acids asparagine, glutamic acid, arginine, threonine and tryptophan in early development. When putting these results into context with other work, it is interesting that the expression of *SlGA3ox1* and *SlDWF4* in tomato fruits are inhibited by the TF JUNGBRUNNEN (JUB), which slows down growth in later stages of fruit development and increases amounts of glutamic acid, aspartic acid and GABA (Shahnejat-Bushehri et al., 2017). Since CES induces *GA3ox1* and *DWF4* expression in *A*. *thaliana*, a conserved function of SlCES during tomato fruit development, would yield larger fruits with decreased levels of certain amino acids such as glutamic acid, which is in line with our results.

Other BR signaling components, when over-expressed also impacted the development and metabolite composition of tomato fruits. *SlBRI1* over-expression promoted fruit ripening and caused an increase in carotenoids, ascorbic acid, soluble sugars and soluble solids (Nie et al., 2017). Over-expression of SlBES1 promoted fruit softening and over-expression of a dominant version of AtBZR1 enhanced carotenoid accumulation (Liu et al., 2014, 2021). Over-expression of SlBIM1a, as well as its *A. thaliana* orthologue AtBIM1, however, repressed fruit growth and produced strongly dwarfed plants in *S. lycopersicum*, showing that BIM1 function in growth promotion in *A.* thaliana is not conserved in tomato, but that SlBIM1 rather acts as a negative regulator of pericarp development (Mori et al., 2021).

Since there was clear evidence that CES function in the control of GA homeostasis and growth is conserved, we aimed to investigate if SlCES over-expression can increase cold stress tolerance by assessing growth and fruit development at a low ambient temperature of 18°C. This showed that the plants were significantly less compromised than wild-type, both in vegetative growth and the ability of fruit set in these conditions. *S. lycopersicum* has a low basal chilling tolerance, with a weak ability to cold acclimate and while it contains CBFs, they activate a much smaller CBF-regulon in response to cold than in *A. thaliana* (Zhang et al., 2004). As opposed to *SlCES* over-expression, which does not cause obvious morphological defects, *CBF* over-expression is not feasible for application, since it induces strong dwarfism in tomato and other plants (Zhang et al., 2004; Achard et al., 2008) and thus alternatives are in demand. An over-expression of the BR biosynthetic gene *SlDWF* had already been shown to improve the chilling tolerance of *S. lycopersicum* (Xia et al., 2018) and here evidence is provided that SlCES is also a factor that is worth considering in breeding strategies, which aim to improve the yield stability of this crop at low temperatures. Since homologues of CES repress immunity in *A. thaliana* (Malinovsky et al., 2014), potential trade-offs of this approach will have to be evaluated.

## Data Availability Statement

The original contributions presented in this study are included in the article/Supplementary Material, further inquiries can be directed to the corresponding author.

## Author Contributions

HS, TC, TW, WR, TL, and BP designed the research. HS, TC, TW, WR, and MP performed the research. BP wrote the manuscript with major contributions from HS. All authors analyzed the data and contributing to finalizing the manuscript.

## Conflict of Interest

The authors declare that the research was conducted in the absence of any commercial or financial relationships that could be construed as a potential conflict of interest.

## Publisher’s Note

All claims expressed in this article are solely those of the authors and do not necessarily represent those of their affiliated organizations, or those of the publisher, the editors and the reviewers. Any product that may be evaluated in this article, or claim that may be made by its manufacturer, is not guaranteed or endorsed by the publisher.

## Supplementary Material

Supplementary Figure 1 Phylogenetic tree of bHLH proteins of *A. thaliana* and *S. lycopersicum*.

Supplementary Figure 2 Levels of GAs of the non-C13-hydroxylation pathway in *SlCESoe* lines. GC-MS measurements of different GAs in aerial parts of 14-day-old plants. The values are given in ng/g fresh weight. The results of four biologically replicates and mean and standard deviations are shown.

Supplementary Table 1 Summary of bHLH proteins of *S. lycopersicum*. Proteins identified in this work and by Wang et al. (2015) and Sun et al. (2015) are compared.
