# Supplementary figures and images for: SlCESTA Is a Brassinosteroid-Regulated bHLH Transcription Factor of Tomato That Promotes Chilling Tolerance and Fruit Growth When Over-Expressed
Source: Front Plant Sci. 2022 Jul 14;13:930805. doi: 10.3389/fpls.2022.930805 (PMC9337221; doi:10.3389/fpls.2022.930805)

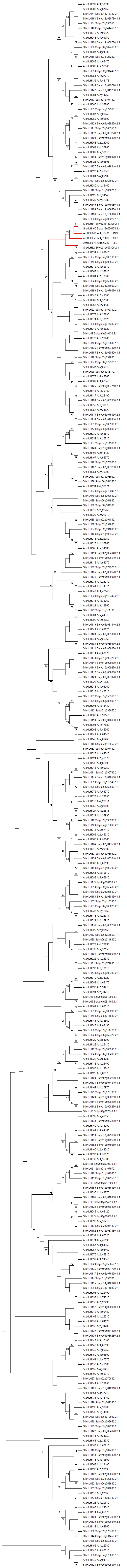

Supplement: Supplementary Figure 1 — Phylogenetic tree of bHLH proteins of A. thaliana and S. lycopersicum. [file Image_1.JPEG]
