# Supplementary material for: SlCESTA Is a Brassinosteroid-Regulated bHLH Transcription Factor of Tomato That Promotes Chilling Tolerance and Fruit Growth When Over-Expressed
Source: Front Plant Sci. 2022 Jul 14;13:930805. doi: 10.3389/fpls.2022.930805 (PMC9337221; doi:10.3389/fpls.2022.930805)
Supplement: Supplementary Figure 2 — Levels of GAs of the non-C13-hydroxylation pathway in SlCESoe lines. GC-MS measurements of different GAs in aerial parts of 14-day-old plants. The values are given in ng/g fresh weight. The results of four biologically replicates and mean and standard deviations are shown. [file Image_2.pdf]

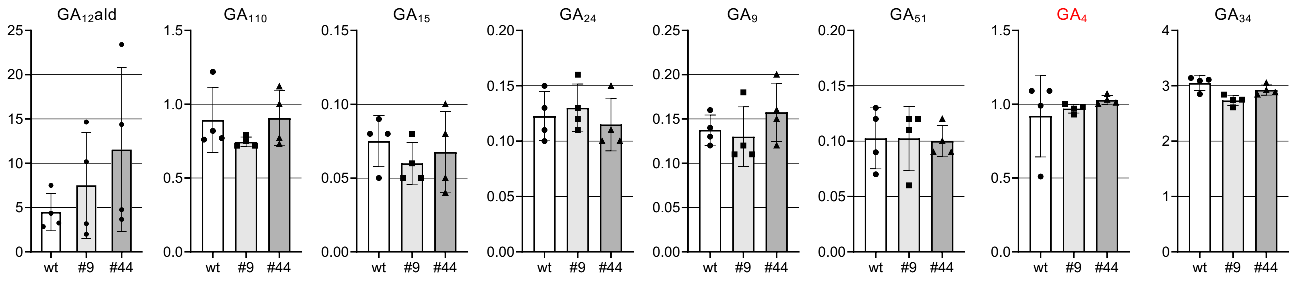

SUPPLEMENTARY FIGURE 2. Levels of GAs of the non C13-hydroxylation pathway in SlCESoe lines.

GC-MS measurements of different GAs in aerial parts of 14-day-old plants. The values are given in ng/g fresh weight. The results of four biologically replicates and mean and standard deviations are shown.
